# Assessment of Influential Variables in the Pricing of Locally Manufactured Pharmaceuticals: Current Challenging Situation in Iran

**DOI:** 10.22037/ijpr.2020.112288.13662

**Published:** 2021

**Authors:** Nikinaz Ashrafi Shahmirzadi, Akbar Abdollahiasl, Abbas Kebriaeezadeh

**Affiliations:** a *Department of Pharmacoeconomics and Pharmaceutical Administration, School of Pharmacy, Tehran University of Medical Sciences, Tehran, Iran. *; b *Pharmaceutical Management and Economics Research Center, Tehran University of Medical Sciences, Tehran, Iran.*

**Keywords:** Pharmaceutical pricing, Influential variables, Government policy, Conflict of interest, Lack of transparency

## Abstract

The lack of transparency and predictability seems to remain one of the major complaints in the pharmaceutical pricing procedure in Iran, but there is not enough official evidence to support it. The main objective of this study was to identify influential variables officially or unofficially influencing the pharmaceuticals pricing in Iran and also clarifying the degree of importance of each variable from the viewpoints of two groups: the pricing Commission members (owners of pricing procedure) and other stakeholders in the pharmaceutical sector. Semi-structured interviews with experts were performed to extract the influential variables. A Likert scale questionnaire was designed based on extracted variables and used in above-mentioned two groups of experts. The validity and reliability of the questionnaire were assessed before use. About 68 influential variables were extracted which classified into eight categories or domains. Less than 50% of extracted influential variables on pharmaceutical pricing have been mentioned in Iran pricing regulations. There were statistically significant differences between the two group’s viewpoints in terms of importance and effect of some variables on pricing procedure. Conflict of interest, lack of transparency and a sound framework were found as the main problems in Iran pharmaceutical pricing procedure and may lead to “case-by-case” decision making. As such, the output of the pricing commission is not transparent and predictable for its beneficiaries.

## Introduction

The Islamic Republic of Iran, with an estimated Gross Domestic Product (GDP) of US$ 447.7 billion in 2017 and a population of about 80.6 million people, is the second-largest economy in the Middle East and North Africa (MENA) region after Saudi Arabia (1). Iran’s current pharmaceutical expenditure includes 25%-65% of the total health expenditure in the private and public sectors like the most developing countries (2, 3).

According to the law related to the regulations on Medical and Pharmaceutical Affairs passed in 1955 and its subsequent amendments, all activities of the pharmaceutical sector, including production, import, distribution, and pricing of the pharmaceuticals, are under the direct supervision of Iran’s Food and Drug Administration (IFDA) (4). Iran’s pharmaceutical policy was formed based on the compulsory generic pharmaceutical policy in the years after the Islamic Revolution (2, 5). But since 2001, in order to establish competition in the domestic pharmaceutical industry and to increase the quality of medicines, this policy has changed to the branded-generic policy (2). A large part of Iran’s pharmaceutical market is currently in the control of the semi-governmental sector, while the private sector accounts for less than 50% of Iran’s pharmaceutical market (5). In any case, the domestic pharmaceutical industry’s share of Iran’s total pharmaceutical market remains about 60%, and the remaining 40% more includes the cost of high-priced, high-tech imported pharmaceuticals (2). All prescriptions and Over-The-Counter (OTC) medicines in Iran’s pharmaceutical market must enter Iran National Drug List (NDL) prior to registration (4). The procedure of entering in this list is performed by the Iran Drug Selection Committee, which includes assessing the quality, efficacy, safety and cost-effectiveness of the medicines (4). The latter has been added to the procedure following the entry of graduates of the pharmacoeconomics discipline to Iran’s pharmaceutical industry over the last few years (5, 6).

In Iran, the pharmaceutical pricing procedure is carried out by the Pharmaceutical Pricing Commission (referred to as “the Commission” in this paper) in IFDA (7). The commission is composed of 5 members in accordance with the above-mentioned law: Deputy Minister and Chief of IFDA, Director General of Division of Pharmaceutical and Narcotic Affairs (DPNA), Ministry of Commerce (MOC) representative, Managing Director of the Odd Governmental Importing Company (this representative is not present at the moment after the dissolution of the company) and a representative of the pharmaceutical industry (8). This commission determines the price of all pharmaceuticals listed in NDL based on several variables set out in pricing regulation publicly issued by IFDA.

According to the World Health Organization (WHO) guideline on country pharmaceutical pricing policies published in 2015, IFDA uses the reference pricing and negotiation method for pricing the imported pharmaceuticals (IPs) and cost-plus method for pricing the locally manufactured pharmaceuticals (LMPs) (9). According to the recent pricing regulation issued by IFDA in February 2018, it appears that the commission has changed the approach of cost-plus pricing to reference pricing for LMPs, especially for high-tech ones (10).

From the stakeholder’s and market participant’s viewpoint, the lack of transparency, predictability and non-binding to regulations are considered as the most important weaknesses of IFDA, especially in pharmaceutical pricing procedure (2, 11). Like other countries, there are several influential variables considered in the pharmaceutical pricing procedure in Iran. However, despite the numerous regulations published in recent years by IFDA, it seems that many of the influential variables in pricing have not yet been addressed in any of them (10). Therefore, despite the efforts made to modify the pricing model in Iran, pharmaceutical market stakeholders cannot properly predict the price of their products and, as a result, their satisfaction goes down, and they often complain about the output of the commission.

The present study was designed to identify influential variables officially or unofficially influencing the LMPs pricing in Iran. The degree of importance of each variable was studied from the viewpoints of the commission’s members (pricing procedure owners) as well as other stakeholders in the pharmaceutical sectors.

## Experimental

A three-step study was designed and performed in order to identify the influential variables in pharmaceutical pricing procedure and evaluating their effects from different stakeholder’s viewpoints.

*Step 1: Identifying influential variables*

In this part of the study, first, all new and old regulations and instructions publicly issued by IFDA were reviewed in order to extract variables officially mentioned in those documents. At the time of the study, we had access to only four regulations for pharmaceutical pricing publicly issued by the IFDA between years 2011 and 2018.

A semi-structured in-depth interview was conducted with a number of IFDA experts who were present and influential in the commission. It aimed to extract variables that were not mentioned directly in the regulations and instructions, but they could be effective in determining the price of LMPs in Iran. Five people who had at least five years of activity experience in the commission were selected. 

The interview guide for the semi-structured interviews was developed by a consensus panel consisting of three experts in the field of Pharmacoeconomics and Pharmaceutical Administration and all questions were reviewed to make sure they are logical and address the things we need to address. After pilot testing the guide, we used it in the study (12). Before interviews, an introductory letter was sent to each expert explaining the aim of the study. Each interview lasted about 1 to 1.5 h. Questions included information about the variables influencing pharmaceutical pricing in the world and in Iran, as well as their experiences during the presence in the commission. According to the responses, it seemed that data saturation was achieved and therefore, no further interview was needed (13). All questions and responses were recorded with permission and then were transcribed, and all variables mentioned in the responses were coded and extracted using the content analysis method and MAXQDA12 software (14). All the extracted variables were categorized and sent to the interviewees again by e-mail for review and final confirmation.

*Step 2: Designing the questionnaires and assessing validity and reliability*

Considering that the effect of each extracted variables on LMP pricing could vary from the viewpoint of different stakeholders, a Likert scale-based questionnaire was designed to evaluate the viewpoints of different stakeholders in this field. The Likert scale for questions included these scores: 1 (very low effect), 2 (low effect), 3 (moderate effect), 4 (high effect), and 5 (very high effect) (15). A separate option (I have no idea) was considered for each question to prevent wrongly choosing the middle option in the case of having no idea (16). 

Both qualitative and quantitative face validity assessments were conducted on the initial version of the questionnaire using seven final participants’ comments in the study (17). Quantitative assessment of the questionnaire was implemented using the “item impact method”. The above-mentioned participants were asked to rank questions in terms of importance based on a 5-point scale from 5 = very important, 4 = important, 3 = somewhat important, 2 = less important, to 1 = not important (18).

In order to evaluate content validity, the questionnaire was evaluated by 12 academic staff in the field of Pharmacoeconomics and Pharmaceutical Administration. These experts were asked to rate each question in terms of “relevance” and “clarity” and the whole questionnaire in terms of “comprehensiveness” (19). There were two sets of response scales: one for relevance, which ranged from 1 (inappropriate) to 4 (completely appropriate) and the other for clarity, which ranged from 1 (ambiguous) to 4 (completely clear). The experts were requested to modify the questions in order to enhance their clarity where needed and suggest removing or adding any questions (20). The response scale for comprehensiveness ranged from 1 (in comprehensive) to 4 (completely comprehensive) (20). The Item-Content Validity Index (I-CVI) with the acceptance level of 78% and the Scale-Content Validity Index (S-CVI) with the acceptance level of 80% were calculated for each question and the entire questionnaire, respectively (21, 22). We also calculated the Inter-rater agreement index for this questionnaire. The minimum acceptable level for this index is usually 70% (20).

The test-retest reliability assessment was carried out by using the final questionnaire and after performing all recommended changes based on the validity assessment. As answers in the Likert scale are considered to be categorical, the weighted Kappa coefficient was used to assess test-retest reliability. Kappa coefficients with the value of 0.41-0.60 indicate ‘moderate’ agreement, 0.61-0.80 indicating ‘substantial’ agreement, and 0.81-1.00 indicating ‘almost perfect’ agreement (23). Finally, Cronbach’s alpha was used to assess the internal consistency of a questionnaire. Often, the alpha coefficient above 0.7 is considered as desirable (24, 25).

*Step 3: Collecting answers to the questionnaire by pharmaceutical Experts*

The validated questionnaire was completed by the selected pharmaceutical experts. Two groups of experts were selected; all of them had at least four years of experience in the field of pharmaceutical industry management: a) those who have had the experience of being present in the commission during their working period (Group A). b) other stakeholders in the pharmaceutical sector who have never been present at the Commission (Group B). 

The questionnaires were sent by e-mail to all participants and responses and comments were also received by e-mail. According to the analyzing method for Likert scale-based surveys, analysis of the responses was performed using both descriptive and inferential statistics. Since valuable information was not obtained from the “median” of raw data, “mode” and “range” were used as a measure of central tendency and measure of dispersion, respectively. Since Likert scale-based variables were discrete and ordinal, data analysis was performed using the Mann-Whitney U test which is a nonparametric test to achieve statistical significance of the differences between responses of two groups of people (A and B) (26).

## Results

*Step 1: Identifying influential variables*

A total of 66 variables influencing the pricing of LMPs were extracted from reviewing regulations and expert opinions. These variables were classified into eight categories or domains in order to facilitate the expression of various variables. The domains were selected based on the discussions conducted by researchers of this study. Since statistical analysis is not performed on these domains separately, the displacement of variables within these domains will not have any effect on the results of the study. These domains and the details of extracted variables are presented in Tables 1 and 2.

*Step 2: Designing the questionnaires and assessing validity and reliability*

The questionnaire was developed based on variables identified in the qualitative study. This questionnaire included 66 questions and was structured in eight defined domains for more convenience in completion. 

Face validity was assessed, and some changes were performed on the structure of the questions so it has more visual appeal. For quantitative assessment of face validity using the item impact method, the participants ranked all questions in terms of importance and all questions were scored more than 1.5 points. This means that in all questions, at least four people gave points 4 or 5 to the questions and the minimum average score for each question was equal to 3, and therefore, all questions remained in the questionnaire. 

In content validity assessment, Item Content Validity Index (I-CVI) and Scale Content Validity Index (S-CVI) of each question and the whole questionnaire, respectively, regarding the relevance and clarity exceeded the acceptable level of 78% for I-CVI and 80% for S-CVI. Only one question with both relevance and clarity I-CVI below acceptable level was eliminated by the research team. The interrater agreement index was about 93.3% for relevance and 83.3% for clarity of questionnaire, and both cases were above the acceptance level of 70%. The comprehensiveness of the questionnaire was calculated to be 91.6%.

For assessing test-retest reliability, all 11 participants answered all questions on both test and retest time. Weighted Kappa coefficients were calculated for each question and all achieved the acceptable level. Therefore, there was no need to change any questions of the questionnaire.

Cronbach’s alpha was calculated for the total instrument. This coefficient was equal to 0.873, so it exceeded the acceptable level. Therefore, it concluded that the questionnaire had acceptable internal consistency and all questions were worthy of being remained in the questionnaire. 

*Step 3: Collecting answers to the questionnaire by pharmaceutical Experts*

Among 59 experts selected for this study, 46 experts finally responded to the questionnaire. Thus, the response rate was equal to 78%. The number of participants in each study group is shown in Table 3.

According to the analyzing method for Likert scale-based surveys, “Mode” and “Range” were calculated for raw data of two groups of study participants (Group A and B) separately so that a comparison can be made between these groups. 

Regarding the “range”, as a measure of dispersion in discrete variables, it can be seen that the answers are at most two levels apart. This does not provide us with so much information, because these two levels can be attributed to the difference between the scores 1 or 5 with 3, or whether the difference between 4 and 2.

In order to determine the variables with the greatest effect on pricing LMPs from Group A and Group B viewpoints, variables were ranked based on the number (%) of people who responded 4 and 5 (High effect and very high effect), in each group A and B. 

Accordingly, the variables determined to have the highest effect (4 and 5) on LMPs pricing from the viewpoint of more than 50% of participants in Group A and B, are presented in Table 4. We can see from the viewpoint of Group A, there are 26 variables, and from the viewpoint of Group B, there are 28 variables, which more than 50% of experts in each group identified them as the most influential variables (4 or 5) in LMPs pricing. Sixteen variables were found to be common between these two groups, as shown by “AB” in the “Groups viewpoint” column of Table 4. Group A and B pointed to 10 and 12 other variables, respectively, which have been considered as the areas of disagreement and shown by letter “A” and letter “B”, respectively, in “Groups viewpoint” column of Table 4. The percentage of those who gave these variables 4 and 5 scores in the opposing groups was less than 50%. It means that less than 50% in the opposing group considered these variables important in the pricing procedure of LMPs. 

The Mann-Whitney U test was performed to determine whether the differences between responses of these two groups were statistically significant. Regarding variables influencing LMPs pricing, in 17 out of 66 questions, there were statistically significant differences between the two groups, and there was no statistically significant difference in the remaining variables. 

As can be seen in Table 4, all variables which were found to be different from the viewpoint of group A and B in primary analysis using “Mode”, showed a significant difference using the Mann-Whitney U test, except 5 variables with the code of D2-9, D4-4, D4-6, D4-11, and D7-8. The p-value of these variables is underlined in Table 4. The direction of the differences can be obtained from the “Groups viewpoint” Column. It can also be concluded that those variables with the code of D2-9, D4-4, D4-6, D4-11, and D7-8 are among the common variables between groups A and B.

According to these data analysis, variables including being an orphan medicine, the number and variety of production steps, being in a drug shortage situation, number of years of price stability, reference basket price, the cost obtained from Pharmacoeconomic studies, exchange rate and its fluctuations, customs duties, financial costs of the company, and general inflation rate (official rate) are among the effective variables in pricing procedure from the viewpoint of Group A experts (the Commission Members). On the contrary, Group B experts believe that the Commission Members place very little importance on these variables when setting a price.

On the other hand, Group B experts believe that variables like the way of presenting a pharmaceutical pricing file by a pricing expert, conflict of interest of members of the commission, personal tastes and subjective preferences of members of the commission, time of presenting a drug pricing file in the commission, physician’s and some association’s lobbying, presence of MOC representative in the commission, and supplier company advertisement before entering the market are among the most effective variables in pricing procedure in the commission. As can be seen, almost all of these variables are categorized in “conflict of interest, personal tastes and subjective preferences” domain.

## Discussion

The main objective of this study was to identify influential variables officially or unofficially influencing the pharmaceuticals pricing in Iran and also clarifying the degree of importance of each variable from the viewpoints of two groups: owners of pricing procedure (the Commission members), and other stakeholders in the pharmaceutical sector. 

Based on the last IFDA pharmaceutical pricing regulation issued in February 2018, It can be said that less than 25 variables out of 66 extracted variables have been mentioned in this regulation to some degree. Therefore, it seems that the commission members (the owners of this procedure) acknowledge that variables beyond the variables mentioned in the various IFDA regulations can be influential in the pricing procedure. This can endorse the lack of clarity of pricing procedure and non-binding to regulations, so the pharmaceutical sector stakeholders cannot predict the output of the commission. However, in its latest Guideline on Country Pharmaceutical Pricing Policies issued in 2015, WHO has clearly stated that policies, procedures, and decisions in the pharmaceutical pricing sectors should be completely transparent, and pricing policies should have a suitable legislative framework and appropriate administrative structure (9).

Regarding many of these variables, although they are not explicitly mentioned in pharmaceutical pricing regulations, their existence is understandable to some extent due to their rational effect on pricing policies. Various reasons can be noted regarding these variables. Perhaps, it is attributed to the fact that each of these variables may only influence on the pricing of some limited pharmaceuticals, so that for some of these variables, there may be only one or two examples. This idea is partly confirmed due to the low importance of these variables in the rest of the study. However, extracting a series of variables related to “conflict of interest” among others can be controversial. Certainly, these variables cannot be denied because these are expressed by people who have had the Commission membership history.

In addition, we found discrepancies between the viewpoints of the procedure owners (Group A) and the procedure stakeholders (Group B) regarding the effect of different variables on the pharmaceutical pricing procedure which can be more challenging. Two methods were used to investigate these discrepancies: using “mode” as a measure of central tendency and using the Mann-Whitney U test. Obviously, the use of the statistical test is of more value for finding significant differences between the two groups, but the tables obtained by the use of “mode” can help in determining the direction of these differences. 

The variables classified in the domain of “conflict of interest, personal tastes and subjective preferences” were found as the main difference between these two groups. Based on the information obtained from “mode”, it can be concluded that, for six variables of this domain with a significant disagreement between the two groups, Group B outlined them as variables with great effect on the pharmaceutical pricing, while group A placed less importance on them. Therefore, from the viewpoint of Group B, it seems that the commission itself is the source of conflict of interest and the owners of the pricing procedure themselves are some kind of stakeholders who decide on a case-by-case basis rather than policymaking at the commission. The lack of robust laws and rules with respect to managing conflict of interest in the Iran MOH is considered as one of the reasons. Iran is among the countries that participated in the WHO Good Governance for Medicines (GGM) program. According to some senior experts idea from the IFDA who have participated in this project, the GGM report of Iran also highlights the challenge of conflict between individuals and structural benefits and the management of these conflicts. This report has never been published by the Iran MOH (27). Conflict of interest is not taken into account as an act of corruption but provides a high potential for corruption. This is clearly stated in the Organization for Economic Co-operation and Development (OECD) Guidelines for Managing Conflict of Interest (27-29). A phenomenon called “Revolving Doors” is regarded as one of the causes of the conflict of interest in the IFDA. This phenomenon is the shift of professionals from the public sector to the private sector and vice versa. This phenomenon can lead to a conflict of interest and the possibility of corruption (30). This phenomenon has been clearly seen among the top managers of IFDA for many years. There are no clear rules for combating this phenomenon in IFDA, while in many countries, mandatory waiting periods are set for these individuals, which are also referred to as “Cooling-off Period” in order to cope with this phenomenon (31). 

The way and time of presenting a pharmaceutical pricing file by the pricing expert at the commission can also be linked to the degree of conflict of interest of the pricing expert (32). The time of presenting a pharmaceutical pricing file in the commission is another challenging variable. If the time of pricing a special pharmaceutical is at the beginning or at the end of a Commission meeting, this can be effective on the final price result. This could be due to the lack of a clear procedure and internal rules and agenda for conducting the Commission meeting, so that at the beginning of the Commission meeting, there would be more time to review the medicine pricing files, while at the end of the meeting, due to the lack of time, many files are only briefly reviewed.

Physicians and some associations lobbying are also among the variables with significant differences between groups A and B. Group B considered this variable more important. In many studies, lobbying has been implicated as a variable in decision making and policymaking, especially in healthcare resource allocation (33-35). The Commission members consider lobbying to be ineffective in pricing procedure, maybe because accepting lobbying can also partly reflect their conflict of interest.

The presence of a MOC representative at the commission can be effective, as expressed from the viewpoint of Group B. The main role of this person is creating a balance between the supply side (manufacturing and importing companies) and the demand side (consumer/patient). Although there is no solid evidence, according to the commission members’ opinion who were interviewed in the qualitative study, this representative often comments in favor of price reduction at the expense of the supplier. Because at most commission meetings, they oppose the price increase, therefore, they can play an effective role in pharmaceuticals pricing.

The disagreements regarding the orphan medicines pricing may be due to the fact that, although these pharmaceuticals are marked in the official NDL of Iran, they have not been mentioned in the last pharmaceutical pricing regulation issued by IFDA (10). Furthermore, there are no separate rules regarding other IFDA regulations, including registration of these pharmaceuticals. Therefore, from the viewpoint of Group B, there is no difference between orphan medicines pricing procedure and other pharmaceuticals. In many countries, there are certain rules and regulations for supporting orphan medicines suppliers, which have been applied for many years (36). The United States was the first country passed the Orphan Drug Act in 1983. some other countries have followed this program are Japan (1993), Singapore (1997), Australia (1998) and the EU (2000) (37, 38).

Unlike Group B, from the viewpoint of Group A, the price obtained from the pharmacoeconomic studies is effective on the final price approved by the commission. Currently, the pharmacoeconomic study is being conducted by the Pharmacoeconomic Committee in IFDA at the time of the pharmaceutical entry into the NDL of Iran. As stated in the recent pharmaceutical pricing regulation issued by IFDA, the price determined by this committee is a basis for pricing in the commission. This controversy can be due to the fact that, this regulation has recently been announced officially, and therefore, people of Group B considered a low effect of this variable on the final pricing at the time of the study (5).

Some variables including the number and variety of production steps, pricing in a drug shortage situation, the number of years of price stability, the reference basket price effect on LMPs, exchange rates and fluctuations, customs duties, financial costs of the company and general inflation rate, are variables which were somewhat considered to be effective in approving the final price by Group A. Although these variables may also be reasonably effective in the pricing procedure from the viewpoint of Group B, given to their understanding of the Commission outcomes, they believe that the Commission members do not pay much attention to these variables practically.

**Table 1:** Classification of extracted variables into 8 domains

Domain Code	Domains
**D1**	Variables related to medicine characteristics
**D2**	Variables related to pricing type and situation
**D3**	Variables related to company characteristics
**D4**	Variables related to Conflict of Interest, personal tastes and subjective preferences
**D5**	Cost-related Variables
**D6**	Variables related to the health system and payment
**D7**	Variables related to economic indicators and market
**D8**	Variables Related to International Treaties

**Table 2:** Details of extracted variables in different domains

Domains	Q. Code^a^	Variables	mentioned In regulation^b^
**D1: Variables related to medicine characteristics**	D1-1	High technology pharmaceuticals	Yes
	D1-2	Under-license manufacturing or technology transfer for the desired medicine	Yes
	D1-3	Orphan medicines	No
	D1-4	Pharmaceuticals for hospital use	No
	D1-5	Narcotics and abusable pharmaceuticals	No
	D1-6	Requiring cold chain	Yes
	D1-7	Number of medicines in the package and the type of packaging	No
	D1-8	The number and variety of production steps	Yes
	D1-9	Special packaging	No
	D1-10	OTC medicines	Yes
**D2: Variables related to pricing type and situation**	D2-1	Reason for pricing	Yes
	D2-2	Being in drug shortage	Yes
	D2-3	Number of years of price stability	No
	D2-4	The Price of Similar imported original brand or branded-generic pharmaceuticals available in Iran's market	Yes
	D2-5	The price of imported competitor original brand or branded-generic pharmaceuticals available in Iran's market	Yes
	D2-6	The price of similar or competitor original brand or branded-generic pharmaceuticals in other market but unavailable in Iran's market	Yes
	D2-7	The price of locally manufactured competitor generic or branded-generic pharmaceuticals available in Iran's market	Yes
	D2-8	The Price of Similar locally manufactured generic or branded-generic pharmaceuticals available in Iran's market	Yes
	D2-9	The Price of Similar locally manufactured generic or branded-generic pharmaceuticals unavailable in Iran's market	No
	D2-10	Reference basket price	Yes
	D2-11	The availability of the desired medicine at competitive prices from other sources (such as parallel imports, passenger goods, *etc.*)	No
**D3: Variables related to company characteristics**	D3-1	Background of the company's activity in Iran's pharmaceutical market	No
	D3-2	The number of manufacturing/import licenses available for the pharmaceutical	Yes
	D3-3	If company is a private or governmental company	No
	D3-4	If company is knowledge-based	No
	D3-5	If company has GMP	Yes
	D3-6	The company's reputation for quality	Yes
	D3-7	The extent to which the owner and key managers of the company are aware or committed	No
Domains	Q. Code^a^	Variables	mentioned In regulation^b^
**D4: Variables related to conflict of interest, personal tastes and subjective preferences**	D4-1	The way of presenting a pharmaceutical pricing file by a pricing expert	No
	D4-2	Conflict of interest of members of the commission	No
	D4-3	Personal tastes and subjective preferences of members of the commission	No
	D4-4	Pharmaceutical knowledge of the people present in the commission	No
	D4-5	Time of presenting a pharmaceutical pricing file in the commission	No
	D4-6	Being aware of the close presence of another domestic manufacturer	No
	D4-7	Current political conditions of the country	No
	D4-8	Subjective or political priorities of the senior managers of the Ministry of Health (MOH)	No
	D4-9	Physician's and some association's lobbying	No
	D4-10	Presence of MOC representative in the commission	No
	D4-11	Media controversy	No
**D5: Cost-related variables**	D5-1	Calculated price by the supplier company	Yes
	D5-2	Calculated price by the pharmaceutical pricing office	Yes
	D5-3	The cost obtained from pharmacoeconomic studies	Yes
	D5-4	Exchange rate and its fluctuations	Yes
	D5-5	Customs duties	Yes
	D5-6	Financial costs of the company	Yes
**D6: Variables related to the health system and payment**	D6-1	Medicine subsidy	No
	D6-2	Insurance coverage	No
	D6-3	Percentage of insurance coverage in outpatient and inpatient care	No
	D6-4	The amount of insurance coverage of the target consumer community	No
	D6-5	Possibility of being prescribed by a specialist or a general practitioner	No
	D6-6	The existence of complementary health insurance in Iran	No
	D6-7	The possibility of a counterfeit type for pharmaceutical or smuggling	No
**D7: Variables related to economic indicators and market**	D7-1	Gross Domestic Product (GDP) of Iran	No
	D7-2	Healthcare expenditure as a share of GDP	No
	D7-3	Pharmaceutical expenditure as a share of health expenditure	No
	D7-4	Income status of target consumer community	No
	D7-5	Market volume and number of target consumers (patients)	No
	D7-6	Total market value	No
	D7-7	Existence of markets for pharmaceutical export	No
	D7-8	Medicine production in the neighboring countries	No
	D7-9	General inflation Rate (official Rate)	Yes
	D7-10	Being aware of supplier discounts in many other pharmaceuticals	No
	D7-11	supplier company Advertisement before entering the market	No
**D8: Variables Related to International Treaties**	D8-1	Main original brand being still patented	No
	D8-2	Being Close to the original brand patent expiration date	Yes
	D8-3	If the medicine included in international conventions and treaties	No

^a^Q. Code: Question Code based on Domain Code.

^b^variables which are mentioned in the last pharmaceutical pricing regulation issued by issued.

^c^N/A: Not Applicable.

**Table 3:** The number of participants in the study in a different group

Groups	Participants in the study	No.
**Experts who were once members of the Commission ** **(A)**	Experts who were a member of IFDA pricing commission at the time of the study	4
	Experts who were a member of IFDA pricing commission, sometime during their working period, but not at the time of the study	11
**Experts who have never been members of the Commission ** **(B)**	Experts who have experience in both fields of the pharmaceutical industry and pharmaceutical importing company management	12
	Experts who have only experience in pharmaceutical industry management.	19

**Table 4:** Variables that, from the viewpoint of more than 50% of Group A and B, have had the highest effect (4 and 5) on LMPs pricing

**Q. Code**	**Variables**	**Groups viewpoint** **(Based on Mode)**	**Asymp. Sig.** **(2-tailed)** ^a^
D1-1	High technology pharmaceuticals	AB^b^	0.243
D1-2	Under-license manufacturing or technology transfer for the desired medicine	AB	0.423
D1-4	pharmaceuticals for hospital use	AB	0.069
D1-10	OTC medicines	AB	0.839
D2-1	Reason for pricing	AB	0.060
D2-4	The Price of Similar imported original brand or branded-generic pharmaceuticals available in Iran's market	AB	0.061
D2-6	The price of similar or competitor original brand or branded-generic pharmaceuticals in other market but unavailable in Iran's market	AB	0.127
D2-8	The Price of Similar locally manufactured generic or branded-generic pharmaceuticals available in Iran's market	AB	0.848
D3-2	The number of manufacturing/import licenses available for the pharmaceutical	AB	0.601
D4-7	Current political conditions of the country	AB	0.062
D4-8	Subjective or political priorities of the senior managers of the MOH	AB	0.674
D5-1	calculated price by the supplier company	AB	0.225
D5-2	calculated price by the pharmaceutical pricing office	AB	0.937
D6-1	medicine subsidy	AB	0.361
D6-2	Insurance coverage	AB	0.635
D7-5	Market volume and number of target consumers (patients)	AB	0.639
D1-3	Orphan medicines	A^c^	0.002
D1-8	The number and variety of production steps	A	0.043
D2-2	Being in drug shortage	A	0.033
D2-3	Number of years of price stability	A	0.008
D2-10	Reference basket price	A	0.041
D5-3	The cost obtained from Pharmacoeconomic studies	A	0.028
D5-4	Exchange rate and its fluctuations	A	0.029
D5-5	Customs duties	A	0.048
D5-6	Financial costs of the company	A	0.032
D7-9	General inflation Rate (official Rate)	A	0.038
D2-9	The Price of Similar locally manufactured generic or branded-generic pharmaceuticals unavailable in Iran's market	B^d^	0.100
D4-1	The way of presenting a pharmaceutical pricing file by a pricing expert	B	0.045
D4-2	Conflict of interest of members of the commission	B	0.029
D4-3	Personal tastes and subjective preferences of members of the commission	B	0.033
D4-4	Pharmaceutical knowledge of the people present in the commission	B	0.426
D4-5	Time of presenting a drug pricing file in the commission	B	0.000
D4-6	Being aware of the close presence of another domestic manufacturer	B	0.149
D4-9	Physician's and some association's lobbying	B	0.011
D4-10	presence of MOC Representative in Commission	B	0.037
D4-11	Media controversy	B	0.089
D7-8	Medicine production in the neighboring countries	B	0.546
D7-11	supplier company Advertisement before entering the market	B	0.048

^a^asymptotic significance, 2-tailed, *p-*value associated with the Mann-Whitney U test. (alpha error is set at 5%).

^b^Common variables that more than 50% of experts in both groups identify them as the most influential variables in pricing procedure, based on "Mode".

^c^Based on "Mode", more than 50% of experts in group A identify these variables as the most influential variables in pricing procedure.

^d^Based on "Mode", more than 50% of experts in group B identify these variables as the most influential variables in pricing procedure.

## Conclusion

Apart from the problems in the commission structure and the pricing procedure in IFDA, it seems that lack of transparency, non-binding to regulations, and lack of a sound framework avoiding the conflict of interest may be considered the bigger problems. As such, the output of the commission is not predictable for its beneficiaries. This is also evident in other procedures in the IFDA. In an editorial paper carried out by Cheraghali, it has been said that in addition to IFDA’s efforts to clarify its policies and procedures, the lack of consistency and commitment to these policies and regulations is the substantial weakness of the IFDA. Therefore, the decision-making would be performed on a “case-by-case” basis and the pharmaceutical companies would receive different answers for the same requests, especially from the pharmaceutical pricing commission (2).

Such “case-by-case” decisions may have some disadvantages; for example, since this decision is more based on expert discussions, the knowledge of the Commission members can be effective in the final decision, and the lack of knowledge can lead to a reduction or increase in the final price and ultimately injustice in the commission’s output (29).

Perhaps one way for clarifying and balancing the commission’s decisions is changing the current arrangement of the commission and considering the presence of representatives from the High Council of Insurance, the Union of Iranian Drug Importers, the Raw Materials Syndicate, and the Iranian Human Pharmaceutical Industry Owners Syndicate. In order to avoid conflict of interest, especially for guild representatives, the commission should formulate policies and develop more comprehensive regulations for the pricing procedure rather than taking case decisions on the pricing of each pharmaceutical.
